# Ferroptosis: a novel regulated cell death participating in cellular stress response, radiotherapy, and immunotherapy

**DOI:** 10.1186/s40164-023-00427-w

**Published:** 2023-07-27

**Authors:** Xiaogang Zheng, Xiaodong Jin, Fei Ye, Xiongxiong Liu, Boyi Yu, Zheng Li, Ting Zhao, Weiqiang Chen, Xinguo Liu, Cuixia Di, Qiang Li

**Affiliations:** 1 Department of Medical Physics, Bio-Medical Research Center, Institute of Modern Physics, Chinese Academy of Sciences, Lanzhou, 730000 China; 2 Key Laboratory of Heavy Ion Radiation Biology and Medicine, Chinese Academy of Sciences, Lanzhou, 730000 China; 3 Key Laboratory of Basic Research on Heavy Ion Radiation Application in Medicine, Lanzhou, 730000 Gansu China; 4 University of Chinese Academy of Sciences, Beijing, 100049 China; 5 Division of Thoracic Tumor Multimodality Treatment and Department of Radiation Oncology, Cancer Center, West China Hospital of Sichuan University, Chengdu, 610041 China

**Keywords:** Ferroptosis, Lipid peroxidation, ER stress, Autophagy, Radiotherapy, Immunotherapy

## Abstract

**Background:**

Ferroptosis is a regulated cell death mode triggered by iron-dependent toxic membrane lipid peroxidation. As a novel cell death modality that is morphologically and mechanistically different from other forms of cell death, such as apoptosis and necrosis, ferroptosis has attracted extensive attention due to its association with various diseases. Evidence on ferroptosis as a potential therapeutic strategy has accumulated with the rapid growth of research on targeting ferroptosis for tumor suppression in recent years.

**Methods:**

We summarize the currently known characteristics and major regulatory mechanisms of ferroptosis and present the role of ferroptosis in cellular stress responses, including ER stress and autophagy. Furthermore, we elucidate the potential applications of ferroptosis in radiotherapy and immunotherapy, which will be beneficial in exploring new strategies for clinical tumor treatment.

**Result and conclusion:**

Based on specific biomarkers and precise patient-specific assessment, targeting ferroptosis has great potential to be translated into practical new approaches for clinical cancer therapy, significantly contributing to the prevention, diagnosis, prognosis, and treatment of cancer.

## Background

Ferroptosis is a novel regulated cell death (RCD) mode caused by damage to lipid membranes due to the accumulation of lipid peroxides and reactive oxygen species (ROS) produced by iron metabolism, which was first observed and named by Stockwell in 2012 [1, 2]. Ferroptosis is driven by lethal lipid peroxidation resulting from imbalances in cellular metabolism and redox homeostasis [2]. More and more studies have shown that ferroptosis is involved in stress processes such as endoplasmic reticulum stress and autophagy, and ferroptosis is associated with many diseases, including degenerative diseases, cancer, acute kidney injury, I/R injury of the heart, liver, and kidney, as well as other diseases such as acute myeloid leukemia, age-related macular degeneration (AMD), psoriasis, and hemolytic disorders [1, 3]. Induction of ferroptosis by experimental small-molecule compounds or clinical drugs is emerging as an effective antitumor strategy for various types of cancer, especially in iron-rich and higher mitochondrial abundance tissues such as hepatocellular carcinoma (HCC), pancreatic ductal adenocarcinoma (PDAC), breast cancer and non-small cell lung carcinoma (NSCLC) [4, 5]. Here we summarize the role of ferroptosis in cellular stress responses, as well as its potential role in cancer radiotherapy and immunotherapy.

## Hallmarks and mechanisms of ferroptosis

### Hallmarks of ferroptosis

Most organisms rely on oxygen as the final electron acceptor in redox-based metabolic processes, and one of the key issues in cell fate determination is the response of cells to oxidative stress. In the process of oxidative stress, irreversible oxidative damage of the lipid membrane bilayer may lead to ferroptosis. Mitochondria are the core sites for aerobic respiration and energy metabolism in mammals. Through the citric acid cycle and oxidative phosphorylation, substrates such as glucose, lipids and proteins are oxidized to produce ATP, which regulates the material cycle and energetic metabolism of organisms. During electron transport and oxidative phosphorylation, some electrons reduce oxygen to form superoxide, a key source of reactive oxygen species (ROS). Imbalance in ROS homeostasis is a critical trigger for lipid peroxidation and ferroptosis induction.

Morphological features of ferroptosis include loss of cell membrane integrity. On the subcellular level, mitochondria usually show morphological abnormalities in the case of ferroptosis. The primary manifestation is the shrinkage of the mitochondrial membrane, resulting in the increase of membrane density and the decrease of inner mitochondrial membrane cristae [1]. In a few cases, the cell morphology becomes rounded, and the number of autophagic vacuoles increases as well.

Different from known cell death modes such as apoptosis, necroptosis, pyroptosis, and cuproptosis, there is usually no change in nuclear morphology, DNA fragmentation, or Caspase signal transmission during ferroptosis, and this process cannot be reversed by the inhibitors of the above-mentioned RCD modes [3, 6, 7]. In addition, ferroptosis induction is usually accompanied by iron and ROS accumulation, lipid peroxidation, and mitogen-activated protein kinase (MAPK) activation. In contrast, when ferroptosis occurs, cystine-glutamate antiporter (system Xc^−^) inhibition, NADPH oxidation, glutathione (GSH) depletion, and loss of mitochondrial membrane potential (ΔΨm) are common [8].

### Mechanisms of ferroptosis

**Fig. 1:**
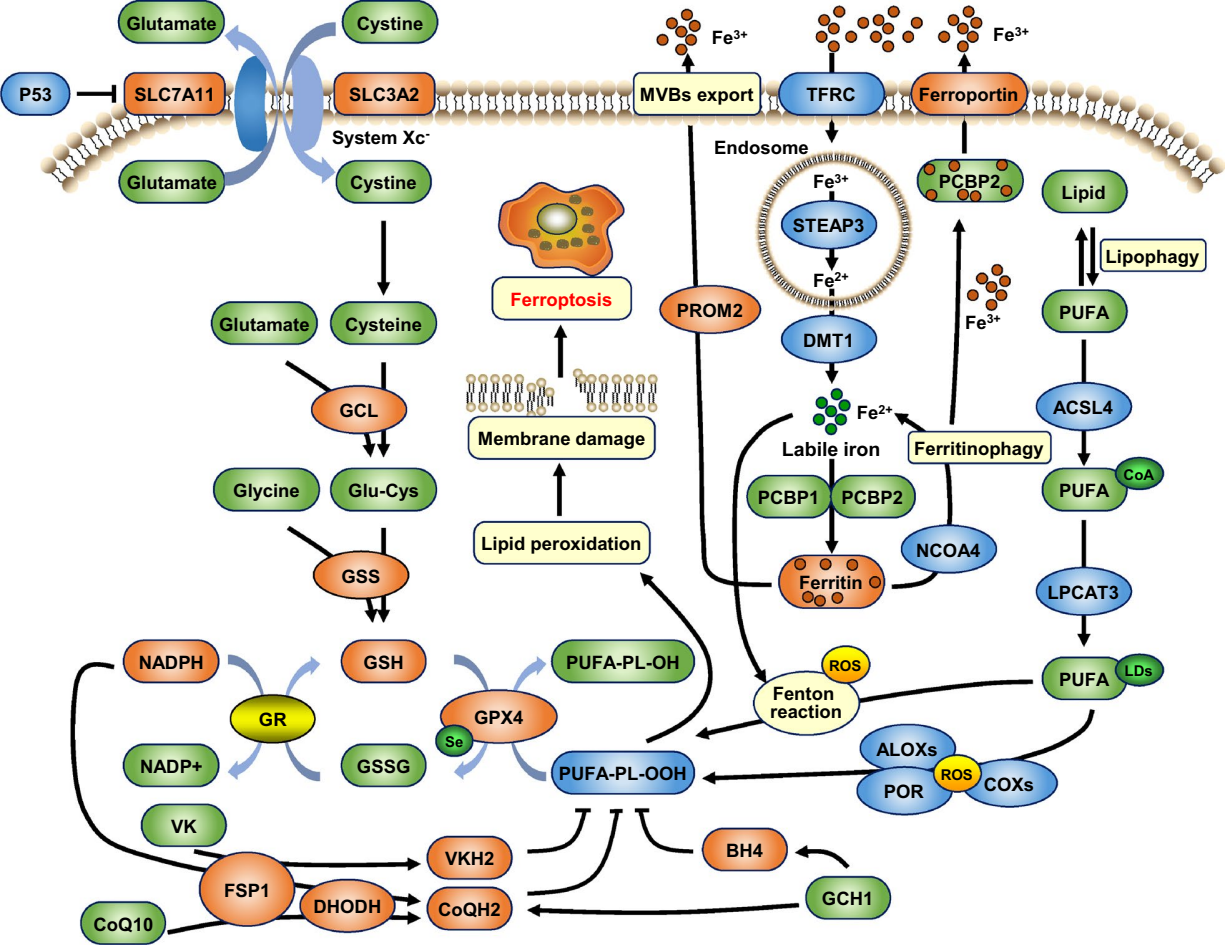
Mechanisms of ferroptosis. Ferroptosis is caused by iron-dependent lipid peroxidation. The extracellular circulating iron ions (Fe^3+^) are transferred into cells via TFRC, catalyzed to Fe^2+^ by the reductase STEAP3 in endosomes, and released into the cytoplasm by DMT1 for cellular processes. The usable iron ions form a labile iron pool or are stored in ferritin. Excess iron ions are translocated into the extracellular circulation by FPN or prominin-2-mediated ferritin-containing MVBs. The Fenton reaction induced by labile iron generates ROS and activates iron-containing enzymes such as ALOXs and POR, resulting in lipid peroxidation and membrane damage. NCOA4-mediated ferritinophagy releases iron from ferritin, leading to membrane oxidative damage and ferroptosis. Cystine is imported into cells by the transmembrane glutamate/cystine antiporter system Xc^−^ and is subsequently oxidized to cysteine. Cells synthesize GSH using cysteine, glutamate, and glycine as substrates under the catalysis of GCL and GSS. Using GSH as a cofactor, GPX4 reduces PUFA-PL-OOH to non-toxic PUFA-PL-OH and exerts antioxidant effects, which alleviates membrane damage caused by lipid peroxidation, thus inhibiting ferroptosis. FSP1-CoQH2, DHODH-CoQH2, FSP1-VKH2, and GCH1-BH4 signaling also inhibit lipid peroxidation-induced ferroptosis. *ACSL4* acyl-CoA synthetase long-chain family member 4, *ALOXs* lipoxygenases, *BH4* tetrahydrobiopterin, *CoQ*_*10*_ coenzyme Q_10_, *DHODH* dihydroorotate dehydrogenase, *DMT1* ferrous ion membrane transport protein 1, *FPN* ferroportin, *FSP1* ferroptosis suppressor protein 1/AIFM2, *GCH1* GTP cyclohydrolase 1, *GCL* glutamate–cysteine ligase, *GSS* glutathione synthase, *GPX4* glutathione peroxidase 4, *GR* glutathione reductase, *GSH* glutathione, *GSSG* oxidized glutathione disulfide, *LPCAT3* lysophosphatidylcholine acyltransferase 3, *MVBs* multivesicular bodies, *POR* cytochrome P450 oxidoreductase, *PROM2* prominin-2, *PUFA* polyunsaturated fatty acid, *PUFA-PLs* polyunsaturated fatty-acid-containing phospholipids, *PUFA-PL-OH* PUFA phospholipid alcohols, *PUFA-PL-OOH* phospholipid with peroxidized polyunsaturated fatty acyl tail, *ROS* reactive oxygen species, *STEAP3* six-transmembrane epithelial antigen of the prostate 3, *TFRC* transferrin receptor, *VK* vitamin K, *VKH2* reduced form of vitamin K

Intracellular iron accumulation and lipid peroxidation are two major biochemical events that induce ferroptosis (Fig. 1). Multiple organelles, including mitochondria, ER, Golgi apparatus, and lysosome, are involved in regulating iron metabolism and redox balance, suggesting that a dynamic signaling network controls and enforces ferroptosis. Ferroptotic cell death requires three conditions [9], including (1) the need for redox-active iron to drive the peroxidation reaction; (2) the need for polyunsaturated fatty acid phospholipids (PUFA-PLs) as substrates for the peroxidation reaction; (3) the malfunction of the homeostatic repair system that needs to remove lipid peroxidation products. In addition, MAPK and autophagy intensity also regulate ferroptosis [10, 11]. Conversely, lipid peroxidation and ferroptosis can be alleviated by maintaining iron homeostasis, scavenging free radicals, repairing peroxidative damage and stress response, and so on [8, 12].

### Iron homeostasis and ferroptosis

Iron is involved in the synthesis of DNA, heme, and Fe–S clusters, the formation of various enzymes such as lipoxygenase (LOX), xanthine oxidase, NADPH oxidase, and the active site of mitochondrial complexes I and III [13, 14]. The extracellular circulating iron exists in the form of ferritin-bound Fe^3+^, which is transferred into cells via transferrin receptor (TFRC), and then delivered to the endosome and catalyzed by the reductase six-transmembrane epithelial antigen of the prostate 3 (STEAP3) into Fe^2+^. SLC11A2/DMT1 releases Fe^2+^ into the cytoplasm and stores it in ferritin or forms a labile iron pool. Ferritin consists of two subunits, light chain 1 (FTL1) and heavy chain 1 (FTH1), with a highly conserved spherical shell three-dimensional structure that accommodates iron in a safe, soluble, and bioavailable form, preventing iron from being oxidized by ROS and causing oxidative damage. Excess Fe^2+^ is converted into Fe^3+^ by the transmembrane exporter ferroportin/SLC40A1 (FPN) and transported to the extracellular circulation [15]. If the iron homeostasis mechanism fails, the Fenton reaction (Fe^2+^ + H_2_O_2_ → Fe^3+^ + HO^−^ + OH·) induced by iron generates ROS and induces the activation of iron-containing enzymes such as LOX, resulting in the damage of protein, lipid, and DNA [16]. The oxidation potential of OH· generated by the Fenton reaction is as high as 2.73 V, which has a strong oxidization ability and plays a significant role in membrane peroxidation.

Epidemiological studies have demonstrated that high dietary iron intake increases the risk of HCC and breast cancer. In vitro studies indicated that silencing TFRC can inhibit Erastin-induced ferroptosis. Deferoxamine (iron chelator) blocks cells from ferroptosis [1], while exogenous iron supplementation promotes ferroptosis induced by Erastin in NSCLC [17]. Iron-regulating proteins ACO1 and IREB2 regulate ferroptosis at the translational level [18]. Increasing the biosynthesis of Fe–S clusters reduces iron concentration and inhibits ferroptosis. Overexpression or inhibition of FPN1 can negatively or positively regulate Erastin-induced ferroptosis in ectopic endometrial stromal cells (EESCs) [19]. In vivo, IREB2 significantly upregulates the expression of FTL1 and FTH1 and inhibits Erastin-induced ferroptosis. Yue et al*.* developed a co-activated catalytic nanoplatform (CACN) that disassembles under the trigger of ATP and releases Fe_3_O_4_ nanoparticles in mouse breast cancer cells (4T1) or human embryonic kidney cells (HEK293), which lead to ferroptosis through the ROS-triggered lipid peroxidation. The disassembly of CACN also accurately releases doxorubicin (Dox), promotes the Fenton reaction and enhances ferroptosis [20]. Mitochondrial enzyme heme oxygenase 1 (HMOX1) is also involved in the regulation of ferroptosis by catalyzing the degradation of heme to release iron, which leads to iron overload and induces lipid peroxidation [21–23]. Overexpression of HMOX1 increases intracellular iron content and sensitizes tumor cells to ferroptosis by the Nrf2-HMOX1 axis. In contrast, knockdown, pharmacological inhibition, or degradation of HMOX1 prevents ferroptosis by reducing iron overload, ROS production, and lipid peroxidation [24–26]. However, another study showed that as a cell stress-induced protective enzyme, slight up-regulation of HMOX1 exerts a protective effect against ferroptosis [27]. Other methods of increasing iron absorption, reducing iron storage, and limiting iron efflux can lead to iron accumulation and promote ferroptosis [18].

### Lipid peroxidation and ferroptosis

Lipid functions include energy storage, signal transmission, and biomembrane formation. Lipid peroxidation is one of the important features of ferroptosis, which often results in oxidative damage to biological membranes (plasma and inner organelle membranes), lipoproteins, and other lipid-containing molecules [28, 29]. PUFA-PLs are the primary substrates for lipid peroxidation. Acyl-CoA synthetase long-chain family member 4 (ACSL4) and lysophosphatidylcholine acyltransferase 3 (LPCAT3) catalyze the esterification and the incorporation of PUFAs into PLs. PUFA-PLs undergo oxygenase (ALOXs)-mediated peroxidation to form PL-PUFA-OOH, leading to ferroptosis. Inhibition of ACSL4 and LPCAT3 expression reduces the intracellular accumulation of lipid peroxide substrates and inhibits ferroptosis [30]. In mouse embryonic fibroblasts, knockout of ACSL4 inhibits lipid peroxidation and ferroptosis, while re-expression of ACSL4 after RSL3 treatment re-sensitizes cells to ferroptosis [31]. LPCAT3 is essential for ferroptosis execution in massive insertional mutagenesis of haploid KBM7 cells [32]. Lipid peroxidation produces a series of primary (lipid hydroperoxides) and secondary oxidation aldehydes, including propanal, hexanal, malondialdehyde (MDA), and 4-hydroxynonenal (4-HNE). Among them, MDA and 4-HNE have higher reactivity and toxicity, and are associated with cancer, diabetes, neurodegenerative and cardiovascular diseases [28]. MDA can exert its mutagenic potential by cross-linking with DNA or proteins [33]. 4-HNE can generate a variety of cytotoxic and genotoxic stresses, including cell cycle arrest, senescence, and programmed cell death (PCD) [28]. Thus, MDA and 4-HNE have become biomarkers of ferroptosis induced by lipid peroxidation due to iron homeostasis imbalance [34, 35]. Cells detoxify the lipid peroxides by blocking the Fenton reaction, scavenging free radicals, repairing damage caused by toxic oxidation products, upregulating the expression of antioxidant nuclear factor E2-related factor 2 (Nrf2), activating heat shock proteins (HSP), and so on [5, 36].

### Redox system and ferroptosis

#### System XC^−^

The system Xc^−^ is a transmembrane glutamate/cystine antiporter located on the cell surface. Structurally, system Xc^−^ consists of two subunits, the light chain SLC7A11 and the heavy chain SLC3A2, which carry out the transmembrane exchange of intracellular glutamate and extracellular cystine [35]. SLC7A11 is the core functional element of the system Xc^−^, which is abundantly expressed in human tumors. Cells synthesize GSH using cystine, glutamate and glycine as substrates under the catalysis of glutamate–cysteine ligase (GCL) and glutathione synthase (GS). GSH acts as a free radical scavenger and a cofactor of GPX4 to alleviate oxidative stress and maintain redox homeostasis [37, 38]. Inhibiting the function of the system Xc^−^ by small-molecule drugs [class I ferroptosis inducers (FINs)] such as Erastin, sulfasalazine, sorafenib, or gene knockout can significantly reduce the production of intracellular GSH, thereby leading to lipid peroxidation and ferroptosis [1, 39].

#### GPX4

The selenoprotein glutathione peroxidase 4 (GPX4) is a core inhibitor of ferroptosis. In humans, the GPX4 gene is located on chromosome 19, which is about 4 kbp in length and consists of 7 exons and 6 introns [40]. The GPX4 gene has three different isomers, namely cytosolic isoform (c-GPX4), mitochondrial isoform (m-GPX4), and nuclear isoform (n-GPX4) [41]. The expression of each isoform is driven by different promoters and may play different roles in regulating signal transduction in different cell death modes. Pharmacological selenium (Se) induces changes in the expression of m-GPX4 and n-GPX4. n-GPX4 may be a necessary isoform induced by Se to counteract lipid peroxidation, and its underlying mechanism is that Se effectively inhibits GPX4-dependent ferroptosis by activating TFAP2c and Sp1-regulated gene expression [42]. With GSH as a cofactor, GPX4 reduces lipid peroxides (L-OOH) to non-toxic lipids (L-OH) so as to inhibit ferroptosis, and this may be hampered by ML210 or RSL3 [8]. GPX4 is essential for the utilization of membranes by living cells to enhance cellular plasticity and to use peroxides as second messengers in redox signaling [43, 44]. Decreased GPX4 activity leads to the accumulation of lipid peroxides (L-OOH) and the formation of lipid free radicals (L-O) catalyzed by iron, thereby resulting in cytotoxicity and cell death. Small molecule inhibitor (class II FINs) RSL3, ML162, ML210, or genetic inhibition of GPX4 leads to increased cytoplasmic ROS and promotes ferroptosis [39, 45]. DMOCPTL, a derivative of the natural product parthenolide, binds directly to the GPX4 and induces its ubiquitination, thereby inducing ferroptosis in triple-negative breast cancer (TNBC) cells [46]. Silencing GPX4 expression in gefitinib-resistant TNBC cells enhances the anti-cancer effect of gefitinib by promoting ferroptosis [47, 48]. In contrast, overexpression of GPX4 reduces cytoplasmic ROS levels and prevents ferroptosis [8, 49–51].

#### Nrf2

The nuclear transcription factor Nrf2 forms a complex with Keap1 (Kelch-like ECH-associated protein 1) and is degraded by the ubiquitination pathway, keeping Nrf2 at a low level under normal physiological conditions. By competitively inhibiting Keap1, p62 prevents the degradation and promotes the nuclear translocation of Nrf2. In the nucleus, Nrf2 forms heterodimerization with Maf and binds to antioxidant response elements (AREs), and activates downstream genes (such as NQO1, HO-1, FTH1, etc.) that regulate iron and ROS metabolism via MT -1G, and thus inhibits ferroptosis induced by Erastin and sorafenib in HCC cells and xenograft models [5]. Activation of Nrf2 increases the degradation of lipid peroxides generated by 12/15-LOX through upregulation of CHAC1 and aldo–keto reductases AKR1C1/3, leading to ferroptosis resistance in melanoma cells. Inhibition of AKRs can re-sensitize drug-resistant melanoma cells to ferroptosis [52]. NRF2 knockdown or inhibition can decrease the expression of GPX4 and enhance the sensitivity of acute myeloid leukemia (AML) cells to ferroptosis inducers. NRF2 inhibition and GPX4 inhibition synergistically induce ferroptosis, effectively reducing the viability of AML cells [53]. On the other hand, recent studies have shown that up-regulation or stabilization of Nrf2 increases intracellular GSH levels, while only moderately inhibiting ferroptosis, because Nrf2 up-regulation results in collateral sensitivity via the expression of multidrug resistance protein 1 (MRP1), which mediates GSH efflux from cells. Thus Nrf2 increases both GSH synthesis and MRP1-mediated GSH efflux. Therefore, the high Nrf2 expression alone is a poor predictor of ferroptosis resistance in some cases, especially when MRP1-mediated GSH efflux counterbalances the protective effects of increased GSH synthesis [54–56].

#### p53

Ferroptosis is considered an endogenous tumor suppressor mechanism downstream of p53 [57]. p53 can occupy the promoter region of the *SLC7A11* gene, transcriptionally repress *SLC7A11* expression, inhibit system Xc^−^ activity, lead to insufficient cystine uptake and GSH synthesis, increase lipid peroxidation, and cause ferroptosis [58–60]. In addition, downregulation of SLC7A11 by p53 promotes the release of ALOX-12, which oxidizes membrane PUFAs, and exerts a pro-ferroptosis effect [61]. Moreover, p53 activates glutaminolysis by upregulating the expression of glutaminase 2 (GLS2) and enhances ALOX-15 by activating spermidine/spermine *N*1-acetyltransferase 1 (SAT1), jointly aggravating ROS-induced lipid peroxidation and promoting ferroptosis [62–64].

In addition to the aforementioned antioxidant systems and mechanisms, reductive coenzyme Q10 (CoQ_10_) produced by extramitochondrial membranes protein ferroptosis suppressor protein 1 (FSP1) and inner mitochondrial membranes protein dihydroorotate dehydrogenase (DHODH), the reduced form of vitamin K (VKH2) generated by FSP1, and tetrahydrobiopterin (BH4) produced by GTP cyclohydrolase 1 (GCH1) are also endogenous antioxidants with a strong ability to reduce lipid peroxidation and resist ferroptosis [39, 65–68]. Hepatic leukemia factor (HLF) promotes ferroptosis resistance by transactivating gamma-glutamyltransferase 1 (GGT1), thus driving the progression and cisplatin resistance of TNBC [69]. A variety of indole-containing natural products (such as DM10, Brucine, DIM), through inducing the increase of Fe^2+^ and lipid ROS accumulation, decreasing GSH levels, and down-regulating the expression of GPX4 and SLC7A11, have been demonstrated to be novel ferroptosis regulators, with the potential to prevent ferroptosis-related cancers [70].

## Ferroptosis and cellular stress response

### Endoplasmic reticulum (ER) stress and ferroptosis

ER is a double-membrane organelle responsible for protein synthesis, folding, maturation, quality control and transport, and for maintaining cellular calcium homeostasis in eukaryotic cells [71]. Any abnormality in the ER protein quality control mechanism triggers a stress state named ER stress [72]. ER-associated degradation (ERAD) is the first line of quality control mechanisms that contribute to the ubiquitination and degradation of unfolded proteins through the proteasome pathway [73]. If ERAD fails, unfolded protein response (UPR) will be triggered to restore the function of the ER by inhibiting protein translation, synthesis, and restoration [74–76]. If the steady-state reconstruction mechanism fails, intense or sustained ER stress will eventually lead to cell death [77]. The imbalance of ROS homeostasis may affect the correct folding of proteins and cause ER stress. Studies have shown that ER stress plays a critical role and might be the cross-link in the interactions between ferroptosis and other types of RCD [35, 78].

ER stress usually acts as a homeostatic protective mechanism that negatively regulates ferroptosis to maintain cell survival. Sorafenib inhibits system Xc^−^ and induces ER stress and ferroptosis [79]. PERK, which serves as one of the transmembrane signaling proteins during UPR, is also associated with the activation of Nrf2 [73]. In addition to the downstream signal of PERK, IRE1 and ATF4 are also deeply involved in the signal crosstalk of ferroptosis and apoptosis. In pancreatic ductal adenocarcinoma (PDAC) cells, Erastin induces phosphorylation of PERK and increases the expression of ATF4, and transcriptionally upregulates system Xc^−^ and HSPA5. HSPA5 forms a complex with GPX4 to prevent the latter from degradation, maintains cellular antioxidant capacity and inhibits ferroptosis. RNAi of HSPA5 or knockdown of ATF4 enhances Erastin-induced ferroptosis significantly [80]. Activation of the PERK-ATF4-HSPA5 pathway was also observed during dihydroartemisinin-induced ferroptosis in glioma cells [81]. In addition, the combined treatment of Erastin, RSL3 and ATF4 inhibitor promote ferroptosis of glioma cells [82].

However, it has also been reported that ER stress may act synergistically with ferroptosis to inhibit cellular activity in some cases. Erastin induces ER stress and activates the eIF2α-ATF4 pathway, and upregulates C/EBP-homologous protein (CHOP) and CHAC1, indicating that ER stress may be involved in the occurrence of ferroptosis. In prostate cancer cells, docetaxel (DTX) combined with overexpression of CHAC1 notably up-regulates GRP78 and CHOP, increasing intracellular lipid peroxidation while decreasing GPX4 protein, which significantly inhibits cell viability [83]. In mouse embryonic fibroblasts, Erastin and artesunate (ART) induce ER stress and promote p53 upregulated modulator of apoptosis (PUMA) expression via CHOP, promoting the synergistic lethal effect of Erastin/ART and tumor necrosis factor-related apoptosis-inducing ligand (TRAIL) [84]. ART induces ER stress and promotes the expression of PUMA through the PERK-eIF2α-ATF4-CHOP signaling axis, and PUMA participates in the synergistic interaction between ferroptosis and apoptosis [78]. In human intestinal epithelial cells (IEC), accumulation of iron and lipid radicals accompanied by the activation of the GRP78-PERK-ATF4-CHOP pathway was observed both in vitro and in vivo, while inhibition of PERK significantly inhibits ferroptosis [85]. These results suggest that the combined induction of ER stress and ferroptosis effectively improves the efficacy of tumor killing, and may be a novel strategy for cancer treatment.

### Autophagy and ferroptosis

The imbalance of ROS homeostasis causes damage to biomacromolecules and organelles, leading to both bulk autophagy and selective autophagy. Ferritin degradation, iron chelation, lysosomal activity, p53 regulation, and the p62-Keap1-Nrf2 pathway are all involved in the activation of ferroptosis-induced autophagy [76] Studies have shown that autophagy plays an essential role in the induction of ferroptosis. Regulation of autophagy is considered to be one of the critical factors in targeting ferroptosis for antitumor therapy [86, 87]. Notably, key signaling markers of ferroptosis, including SLC7A11, GPX4, Nrf2, p53, HSPB1, CISD1, FANCD2, and ACSL4, are potential regulators of autophagy [86]. Conversely, lipid peroxides promote the formation of autophagosomes, while GPX4 over-expression inhibits ROS-mediated autophagy [88].

### Macroautophagy in ferroptosis induction

Beclin-1 is involved in the ferroptosis-apoptosis interaction in human colon cancer cells, as evidenced by the fact that when co-treated with Erastin and TRAIL, Beclin-1 is cleaved by TRAIL-activated Caspase-8, leading to PUMA activation and driving apoptosis [89]. Beclin-1 can be phosphorylated by AMPK at Ser90/93/96 and forms the Beclin1-SLC7A11 complex, which inhibits the activity of system Xc^−^ and promotes lipid peroxidation. Knockdown of Beclin-1 attenuates the inhibitory effect on the system Xc^−^ of FINs (such as Erastin, sulfasalazine, and sorafenib) both in vitro and in vivo [90, 91]. Inhibition of CDGSH iron-sulfur domain 2 (CISD2) gene promotes Beclin-1involved autophagy, leading to iron accumulation, and enhances sorafenib-induced ferroptosis in HCC cells [92]. Deletion of pirin iron-binding nuclear protein (PIR) initiates autophagy through the binding of Beclin-1 to HMGB1, activates ACSL4 and promotes ferroptosis in human pancreatic cancer cells [93]. RSL3 promotes ferroptosis by mTOR inhibition and autophagy-dependent GPX4 degradation [94].

In acute lymphoblastic leukemia, knockdown of ATG5/ATG 7 or 3-MA treatment inhibits ferroptosis by blocking Hydnocarpin D (HD)-induced autophagy [95]. In malignant mesothelioma cells, NTP-activated Ringer’s lactate (PAL) induced ferroptosis is accompanied by autophagy features such as increases in LC3B-II, p62, and LAMP1 at the early stage [96]. Fingolimod (FTY720) induces ferroptosis and autophagy through the PP2A/AMPK pathway, and ferroptosis and autophagy promote each other and inhibit multiple myeloma effectively [97]. HMGB1 activates MAPKs (including MAPK/JNK and MAPK/p38) by up-regulating the expression of TFRC and positively regulates Erastin-induced ferroptosis in leukemia cells [98]. ATPR, a novel all-trans retinoic acid (ATRA) derivative, induces autophagy and regulates iron homeostasis to trigger ferroptosis in AML both in vivo and in vitro [99]. Inhibition of autophagy flux leads to accumulation of p62, promotes degradation of Keap1 and nucleus translocation of Nrf2, upregulates iron and ROS metabolism-related genes, and exerts anti-ferroptosis effect in HepG2 cells [100, 101]. The autophagy inhibitor chloroquine and the iron chelator deferoxamine attenuate Cd-induced lipid peroxidation and ferroptosis [102].

Some natural products also regulate ferroptosis by inducing autophagy. Amentoflavone (AF) activates the AMPK/mTOR-autophagy signaling pathway and triggers ferroptosis in glioma in vitro and in vivo [103]. Typhaneoside (TYP) triggers autophagic degradation of ferritin by activating AMPK signaling, resulting in ROS accumulation and ferroptosis in AML [104]. Quercetin induces transcription factor EB (TFEB)-mediated lysosomal activation and degrades ferritin, promoting ferroptosis [105]. In NSCLC, curcumin-induced autophagy leads to iron overload and mitochondrial membrane rupture, as well as up-regulation of ACSL4 and down-regulation of SLC7A11 and GPX4, resulting in GSH depletion and lipid peroxidation, ultimately leading to ferroptosis. Either chloroquine or siBeclin-1 attenuates curcumin-induced autophagy and subsequent ferroptosis [106]. 6-Gingerol inhibits the growth of NSCLC and solid tumors by inhibiting the expression of USP14 and promoting autophagy-dependent ferroptosis [107].

Nanomedicine-induced autophagy triggers ferroptosis as well. Iron-based nanomaterials can release Fe^2+^ or Fe^3+^ in acidic lysosomes and generate highly toxic ROS through the Fenton reaction [108]. Synthesized ultrasmall iron oxide nanoparticles (USIONPs) significantly upregulate ferroptosis in glioblastoma (GBM) cells via Beclin-1/Atg5-dependent autophagy. Overexpression of Beclin-1/ATG5 significantly promotes autophagy-induced ferroptosis, while autophagy or lysosome inhibitors can reverse autophagy-induced ferroptosis effectively [109]. In an in vitro and in vivo study, nanoparticle ferritin-bound Erastin and rapamycin (NFER) induce ferroptosis by down-regulating GPX4 and promoting lipid peroxide accumulation. Rapamycin potentiates autophagy-related ferroptosis and inhibits tumor progression [110]. MnO_2_@HMCu_2_-xS nanocomposites (HMCMs) exhibit photothermal (PT)-enhanced GPX4 inactivation and ferroptosis induction. Mn^2+^ generates ROS through the Fenton reaction and promotes the accumulation of lipid peroxides, while rapamycin promotes autophagy and further promotes the process of ferroptosis in human breast cancer models [111].

On the contrary, a few studies reported that autophagy inhibits ferroptosis activity, and inhibition of autophagy flux contributes to the induction of ferroptosis. Superparamagnetic iron oxide nanoparticles (SPIONs) lead to decreased autophagy activity but activate ferroptosis, thereby inhibiting the proliferation, invasion, drug resistance, and tumorigenicity of human ovarian cancer stem cells (HuOCSCs) [112]. In glioblastoma stem cells and neurodegenerative lysosomal storage diseases (LSD), inhibition of autophagic flux and autophagosome accumulation may lead to ferroptosis, suggesting an antagonistic relationship between autophagy and ferroptosis [10, 113]. As we know, low levels of autophagy help to remove damaged organelles and biomolecules and maintain cell survival, while intense and continuous autophagy usually causes autophagic cell death, namely type II PCD. Similarly, the regulation of autophagy on ferroptosis may also be related to the intensity of autophagy. Within a certain threshold, autophagy tends to inhibit ferroptosis, once the threshold is exceeded, autophagy switches to induce ferroptosis, thus playing an antitumor effect.

### No evidence of a significant association between ferroptosis and macroautophagy

There are also studies showing that there is no apparent correlation between autophagy and ferroptosis, and their coexistence in space and time may just be a “coincidence”. The combination of siramesine and lapatinib induces both ferroptosis and autophagic death in breast cancer, but there is no evidence of a correlation between the two processes [114]. Chinese herbal medicines thymus vulgaris and arctium lappa inhibit the proliferation of leukemia and multiple myeloma cells by inducing autophagy, ferroptosis, and apoptosis. However, whether there is a direct relationship between autophagy and ferroptosis remains uncertain [115]. Betaine alkaloid ungeremine induces ferroptosis, autophagy, necrosis, and apoptosis in nine cancer cell lines by activating Caspases, altering mitochondrial membrane potential and increasing ROS, but the relationship between these cellular processes is not clear [116]. Autophagy, ferroptosis, anoikis, necrosis and ER stress all participate in the regulation and transduction of melanoma cell death, but the signaling crosstalk between autophagy and ferroptosis remains unclear [73]. In addition, autophagy activation and ferroptosis induction were also observed in some non-neoplastic diseases, such as heart failure and intracerebral hemorrhage, but there is no solid evidence of a connection between these two physiological processes [117, 118]. Therefore, more studies are still needed to elucidate the crosstalk mechanism between ferroptosis and autophagy for further understanding the function of ferroptosis in physiological processes.

## Selective autophagy in ferroptosis induction

Selective autophagy, including nuclear receptor coactivator 4 (NCOA4) promoted ferritinophagy, RAB7A-dependent lipophagy, mitophagy, clockophagy, and chaperone-mediated autophagy (CMA), all play an important role in the modulation of ferroptosis [4].

### Ferritinophagy

Ferritin can store up to 4500 iron atoms, which effectively avoids oxidative damage caused by iron overload in cells [98]. Ferritinophagy is a selective autophagy process that releases free iron from ferritin, leading to oxidative damage and ferroptosis via the Fenton reaction [119]. Knockdown of *Atg5* inhibits Erastin-induced ferroptosis by reducing intracellular ferrous levels, suggesting that autophagy plays a critical role in ferritin degradation and ferroptosis induction [120, 121].

NCOA4-mediated ferritinophagy is the link between autophagy and ferroptosis [88, 121]. NCOA4 serves as a cargo receptor that targets ferritin to the lysosome and releases free iron by ferritinophagy. Knockdown of *NCOA4* inhibits ferritin degradation and ferroptosis, whereas overexpression of *NCOA4* increases ferritin degradation and promotes ferroptosis. In mouse embryonic fibroblasts, human pancreatic cancer cell lines (PANC1, PANC2.03) and human fibrosarcoma cell line (HT-1080), *NCOA4* deficiency or Atg5 and Atg7 knockdown decreases intracellular labile ferrous and lipid peroxidation, thereby inhibiting Erastin-induced ferroptosis [120, 122, 123]. In glioblastoma, the knockdown of coatomer protein complex subunit zeta 1 (COPZ1) increases the expression of NCOA4, resulting in autophagic degradation of FTH1 and increased intracellular ferrous levels, thus leading to lipid peroxidation and ferroptosis [124].

In addition to the NCOA4-centered signaling axis, other signaling pathways also participate in regulating ferritinophagy. Erastin leads to ferroptosis by autophagic degradation of ferritin and induction of TFRC expression. Defects in autophagy genes *BECN1* and *LC3B* effectively inhibit Erastin-induced ferroptosis [125]. Dihydroartemisinin promotes ferritinophagy by regulating the AMPK-mTOR-p70S6k signaling pathway, which supports the labile iron pool and ROS accumulation, resulting in the induction of ferroptosis in AML cells. Conversely, inhibition of autophagy suppresses ferroptosis in AML cells [126]. p62 promotes autophagic degradation of FPN and ferroptosis in cultured cells and xenograft mouse models [127]. In pancreatic MIN16 cells, NaAsO_2_-induced ferroptosis is dependent on mitochondrial reactive oxygen species (MtROS)-associated ferritinophagy [128]. Signal transducer and activator of transcription 3 (STAT3) mediates the expression and release of cathepsin B (CTSB), which is essential for lysosomal degradation of ferritin and induction ferroptosis in human pancreatic ductal adenocarcinoma (PDAC) cells [129]. Both testosterone and cisplatin can induce ferritinophagy-dependent ferroptosis, and the blockade of autophagy significantly alleviates ferroptosis [130, 131].

### Lipophagy

Lipophagy is a type of selective autophagy that degrades lipid droplets (LDs) substrates by the lysosome. Free fatty acids are available as raw material for mitochondrial β-oxidation, and can be esterified to triglycerides and cholesterol to form LDs. Studies have shown that GTPase RAB7A mediates autophagic degradation of LDs increases the level of intracellular free fatty acids, and promotes RSL3-induced ferroptosis in hepatocytes. Knockout of *RAB7A* or *Atg5* inhibits LDs degradation and reverses RSL3-induced ferroptosis in vivo and in vitro [132, 133]. Conversely, inhibition of lipophagy reduces lipid peroxidation substrates and prevents ferroptosis. Another study showed that genetically enhanced *TPD52* promotes LDs formation, thereby decreasing free fatty acid levels and preventing lipid peroxidation-induced ferroptosis in hepatocytes [134].

### Chaperone-mediated autophagy

CMA is also involved in the execution of ferroptosis. It has been shown that Erastin increases the level of lysosome-associated membrane protein-2a (Lamp-2a) and promotes CMA, which in turn promotes GPX4 degradation and stimulates ferroptosis, while inhibition of CMA stabilizes GPX4 and reduces ferroptosis [135]. HSC70 can also mediate CMA by interacting with Lamp-2a, knockout of HSPA8/HSC70 and Lamp-2a restores GPX4 levels and alleviates Erastin-induced ferroptosis [135, 136].

### Clockophagy

Mechanistically, the core circadian clock regulator aryl hydrocarbon receptor nuclear translocator-like (ARNTL) inhibits ferroptosis by inhibiting the transcription of *Egln2* and activating the pro-survival transcription factor HIF1A. In vivo and in vitro experiments on human fibrosarcoma cells have shown that FINs (RSL3 and FIN56) evoke p62-dependent autophagic degradation of ARNTL, negatively regulating HIF1 and preventing HIF1A-dependent fatty acid uptake and lipid storage, which promotes lipid peroxidation and eventually ferroptosis [137]. Another study showed that the knockout of *SQSTM1* prevents the degradation of ARNTL by clockophagy, thereby exerting an anti-ferroptosis effect [138].

### Mitophagy

Eukaryotic mitochondria generate most of the energy (ATP) through oxidative phosphorylation, which maintains the material circulation and energy metabolism of cells. Mitochondria are also major sites of ROS production if their structure is damaged, thus mitochondria are involved in regulating various forms of RCD [123, 139]. Fortunately, mitophagy can break down dysfunctional mitochondria, maintain ROS homeostasis and prevent apoptosis and lipid peroxidation-related ferroptosis [10, 11]. However, there is also a study showing that BAY 87-2243 induces mitophagy-dependent escalation of lipid ROS and ferroptosis in BRAF (V600E) melanoma cells both in vitro and in vivo [140]. Therefore, the role of mitophagy in the regulation of ferroptosis still needs to be further explored.

## Role of ferroptosis in radiotherapy

### Biological effects of ionizing radiation

Ionizing radiation directly causes DNA damage through the photoelectric effect and the Compton effect. Single-strand breaks (SSBs) can easily be repaired by the damage repair machinery in cells, while double-strand breaks (DSBs) usually lead to cell death, which is the core mechanism of ionizing radiation to kill tumors in the traditional sense. Another aspect that cannot be neglected is that ionizing radiation acts on water molecules to produce unstable free radicals, which causes damage to biological macromolecules such as DNA and protein through indirect effects. Ionizing radiation also activates autoimmunity, with potential advantages in combination with immunotherapy [141].

With the deepening of research, it has been found that ionizing radiation induces ferroptosis in apoptosis-resistant cells, or simultaneously induces mixed-RCD, including both apoptosis and ferroptosis, which will undoubtedly be more helpful in killing tumors and improving the tumor-suppressive effect [142]. Ionizing radiation causes water radiolysis to produce hydroxyl radicals (·OH), a highly reactive oxidant that serves as the main inducer of phospholipid peroxidation. By altering the intake and excretion of iron through FPN and TFRC, ionizing radiation also regulates iron metabolism and homeostasis [51]. The accumulation of labile iron in the cytoplasm produces a Fenton reaction with radiation-induced hydrogen peroxide (Fenton’s reagent), further increasing the level of ·OH. ·OH reacts with DNA, lipids, carbohydrates, and proteins, leading to DNA breaks, protein oxidation and glycation, and ferroptosis finally.

### Radiotherapy induces ferroptosis in cancer cells through a variety of mechanisms

**Fig. 2:**
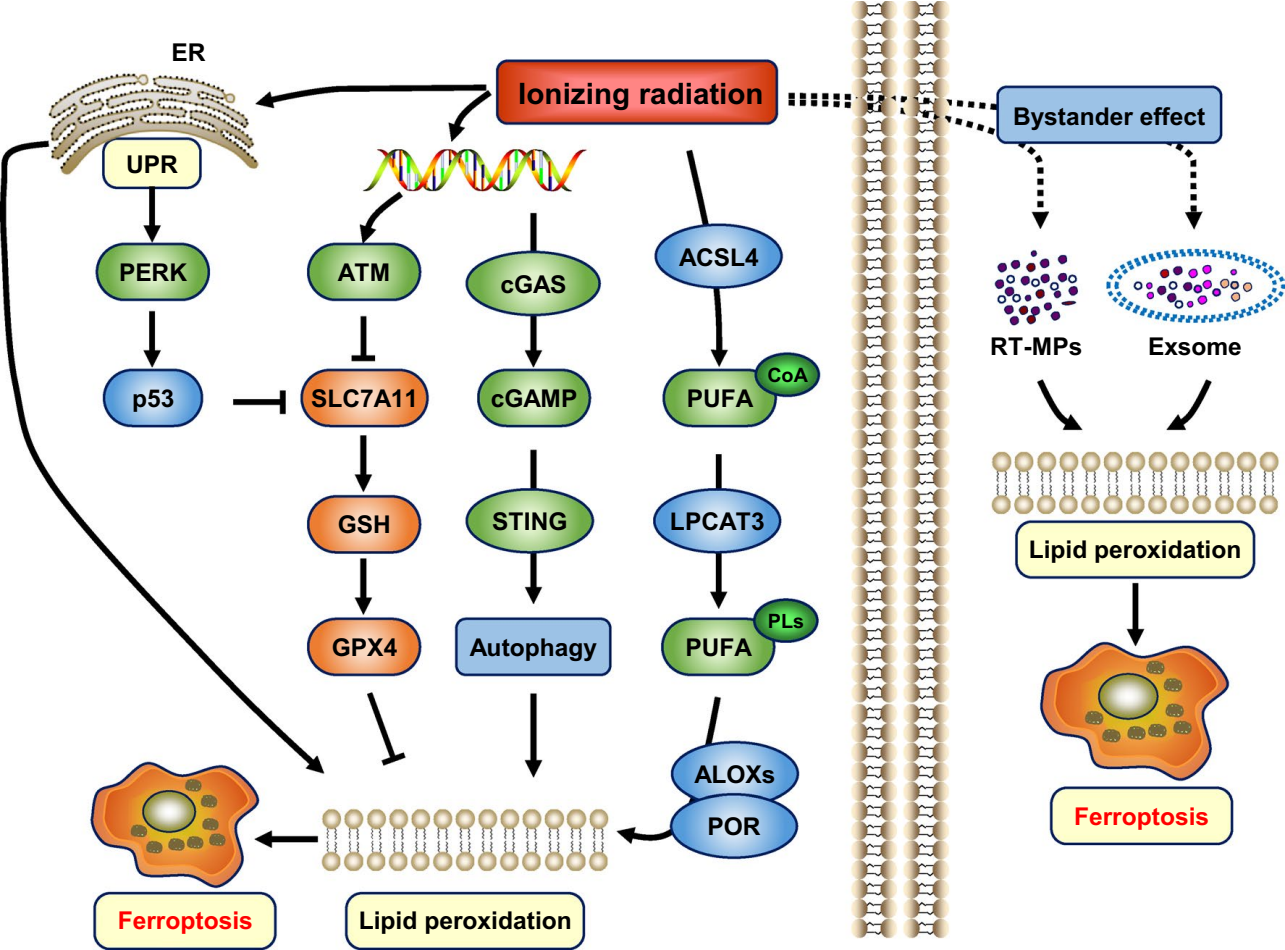
Role of ferroptosis in radiotherapy. Ionizing radiation can cause ferroptosis in several ways. DNA damage induced by ionizing radiation activates ATM and inhibits SLC7A11. DNA damage also activates the cGAS-STING signaling pathway and induces autophagy. Ionizing radiation induces ACSL4 expression and promotes the formation of lipid peroxidation substrates PUFA-PLs. Ionizing radiation causes ER stress, promotes p53 expression through PERK, and transcriptionally inhibits SLC7A11. Ionizing radiation also induces lipid peroxidation in adjacent cells through bystander effects by releasing RT-MPs and exosomes into the tumor microenvironment. *ATM* ataxia telangiectasia mutated protein, *cGAS* cyclic GMP–AMP synthase, *STING* stimulator of interferon genes protein, *PERK* PKR-like ER kinase, *RT-MPs* irradiated tumor cell-released microparticles

Up to now, the basic and applied research on radiation-induced ferroptosis mainly focuses on sparse ionizing radiations such as X- and γ-rays. Several studies have reported that ionizing radiation decreases the levels of SLC7A11, GSH, and GPX4 in cells [143, 144]. One of the mechanisms is that ionizing radiation activates cyclic GMP-AMP synthase (cGAS) signaling, which leads to autophagy-dependent ferroptosis [12] (Fig. 2). Another mechanism is radiation-induced ROS activates ATM and ACSL4 expression, then ATM mediates SLC7A11 downregulation, and ACSL4 increases PUFA-PLs formation for lethal lipid peroxidation. Therefore, ACSL4 ablation eliminates ionizing radiation-induced ferroptosis and promotes radioresistance [145]. In addition, ionizing radiation also induces tumor cells to release irradiated tumor cell-released microparticles (RT-MPs) and exosomes, thereby causing ferroptosis through the bystander effect. Interestingly, there is also evidence that the expression of SLC7A11 and GPX4 induced by ionizing radiation is a stress response, suggesting that oxidative stress caused by radiation serves as a double-edged sword, whether cells ultimately achieve homeostasis reconstruction or heading for ferroptosis, depending mainly on the extent and duration of membrane lipid damage.

Dense ionizing radiation such as heavy ions (especially carbon ions) has physical and biological advantages and exhibits excellent tumor-killing performance, and thus is more suitable for the treatment of locally non-proliferating tumors, tumors that are resistant to conventional radiation, and tumors located near critical tissues and organs that are difficult to surgically remove, especially chordoma, lung cancer, liver cancer, prostate cancer, bone and soft tissue sarcoma, etc. [141, 146–149]. In addition, heavy ions cause cell death in various ways, inducing apoptosis, necrosis, autophagy, premature aging, accelerated differentiation, bystander effects, and ferroptosis, thus exerting a significant role in tumor inhibition. However, there still are relatively few studies focused on heavy ion-induced ferroptosis. Our recent study showed that ionizing radiation activates ER stress and induces ferroptosis accompanied by apoptosis through the PERK and p53-involved pathways, and that mixed-RCD plays a significant role in cancer suppression [150].

### FINs enhance the radiosensitivity of cancer cells in vivo and in vitro

Inactivation of SLC7A11 or GPX4 by FINs (including sulfasalazine, RSL3, ML162, FIN56) enhances the radiosensitivity of cancer cells and xenograft tumors to X-rays, and usually portends a better response and prognosis after radiotherapy [145]. Erastin inhibits GPX4 expression and enhances the radiosensitivity of breast, cervical, and NSCLC to X-rays by inducing ferroptosis [151, 152]. Erastin also increases the sensitivity of breast cancer cells to γ-rays. In human lung adenocarcinoma and glioma patient models, FINs (IKE, RSL3, Sorafenib) and radiation induce ferroptosis synergistically, enhancing the tumor suppression effect of ionizing radiation [4]. Itraconazole enhances the radiosensitivity of nasopharyngeal carcinoma by inducing ferroptosis. In addition, overexpression of Collectrin (CLTRN) promotes ferroptosis by down-regulating GPX4, SLC7A11, and FTH1, thereby enhancing the radiosensitivity of HCC cells to X-rays both in vitro and in vivo [153]. These results suggest that FINs-induced ferroptosis act synergistically with ionizing radiation-induced apoptosis, providing new theoretical support for clinical medicine combined with radiotherapy in treating tumors.

## Role of ferroptosis in immunotherapy

### Cytotoxic T lymphocytes-driven immunity induces ferroptosis

**Fig. 3:**
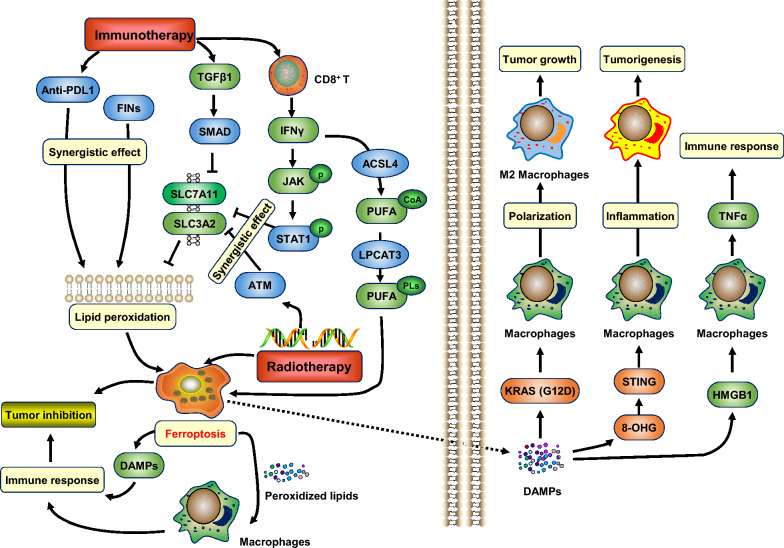
Role of ferroptosis in tumor immunity. Immunotherapy-activated CD8^+^ T cells release IFN-γ, which transcriptionally inhibits SLC7A11 and SLC3A2 by activating JAK1-STAT1 signaling. IFN-γ also induces ACSL4 expression and promotes the generation of lipid peroxidation substrate PUFA-PLs. Immunotherapy-induced release of TGFβ1 transcriptionally represses SLC7A11 through the SMAD-involved pathway. Immunotherapy inhibits SLC7A11 synergistically with radiotherapy-activated ATM. Immunotherapy can also synergize with FINs to promote lipid peroxidation. DAMPs released by ferroptotic cells enhance antitumor immune responses by reversing the immunosuppressive microenvironment. Lipid metabolites activate macrophages immune responses and adaptive immunity-driven cytotoxic T lymphocytes. Furthermore, the release of HMGB1 during ferroptosis leads to the up-regulation of TNFα in macrophages and sustains the immune response. On the other hand, ferroptotic cells can inhibit antitumor immunity by releasing KRAS (G12D), which triggers M2 macrophage polarization. 8-OHG released by ferroptotic cells activates STING signaling in macrophages, leading to an inflammatory tumor microenvironment and causing tumorigenesis. *TNFα* tumor necrosis factor-alpha, *DAMPs* damage-associated molecular patterns, *HMGB1* high mobility group protein B1, *8-OHG* 8-hydroxyguanosine, *IFNγ* interferon-gamma, *TGFβ* transforming growth factor β, *PD-L1* programmed cell death-ligand 1, *KRAS (G12D)* Kirsten rat sarcoma viral oncogene homolog-G12D mutation

Immune checkpoint blocking therapy has become one of the most prevalent cancer treatments in recent years, and the immune system can suppress tumors to some extent by inducing ferroptosis. Ferroptosis promoted by cytotoxic T lymphocytes is a potential antitumor mechanism, and targeting ferroptosis synergized with immune checkpoint inhibition is a potentially effective cancer therapy strategy [154]. In ovarian cancer and melanoma-bearing mouse models, interferon (IFN)-γ released by immunotherapy-activated CD8^+^ T cells down-regulates the expression of SLC7A11 and SLC3A2, inhibits cystine uptake in tumor cells, promotes lipid peroxidation and ferroptosis, thereby increasing the antitumor efficacy of immunotherapy (Fig. 3). In vivo, cystine/cysteine depletion combined with checkpoint blockade synergistically enhances T cell-mediated antitumor immunity and induces tumor cell ferroptosis [155]. Another study showed that IFN-γ inhibits the activity of system Xc^−^ via JAK-STAT signaling, increasing ROS levels and reducing mitochondrial membrane potential, sensitizing HCC cells to ferroptosis [156]. Nivolumab increases IFN-γ while decreasing SLC3A2 expression, exhibiting a better clinical prognosis of cancer. Hemoglobin plus photodynamic therapy (PDT) treatment releases IFN-γ from T lymphocytes, enhancing the sensitivity of tumor cells to ferroptosis and promoting tumor suppression [157]. It's worth noting that immunotherapy also sensitizes tumors to radiotherapy by promoting ferroptosis, which sheds light on a new treatment approach for tumors that combines radiotherapy with immunotherapy. Mechanistically, IFN-γ derived from immunotherapy-activated CD8^+^ T cells and radiotherapy-activated ATM synergistically inhibits SLC7A11, resulting in decreased cystine uptake and enhanced lipid peroxidation, and subsequently leading to ferroptosis to improve tumor suppression [143, 155].

### Ferroptosis regulates antitumor immunity

With the increase of ferroptosis-related studies, it has been realized that ferroptosis is immunogenic and can activate innate immune [158, 159]. Moreover, damage-associated molecular patterns (DAMPs) or lipid metabolites released by ferroptotic cells activate the immune responses of adjacent immune cells and adaptive immunity-driven cytotoxic T lymphocytes, thus confirming that ferroptosis is a type of immunogenic cell death (ICD) [158, 160–162]. Ferroptotic cells induce ICD by releasing ATP and HMGB1, or by regulating HSP70 and HSP90, contributing to the suppression of colon cancer [163]. The release of HMGB1 during ferroptosis leads to the up-regulation of tumor necrosis factor-alpha (TNFα) in macrophages and sustains the immune response [164]. In murine glioma or fibrosarcoma cells, ATP and HMGB1 released by ferroptotic cells can be phagocytosed by bone-marrow-derived dendritic cells (BMDCs), which subsequently mature to produce IL-6 and promote tumor suppression [165]. RSL3-induced ferroptosis leads to ICD in an ATP and P2X7-dependent manner, which promotes phenotypic maturation of BMDCs, and produces vaccine-like effects in immunocompetent mice [166, 167].

SAPE-OOH (1-Stearoyl-2′-15-HpETE-sn-glycero-3-phosphatidylethanolamine), a phospholipid that acts as an ‘eat-me’ signal on the surface of ferroptotic cells, causing TLR2-mediated phagocytosis in mouse mammary tumor [168]. In mouse xenografts of head and neck squamous cell carcinoma (HNSCC), ferroptosis significantly reduces the number of myeloid-derived suppressor cells (MDSCs) and tumor-associated M2-like macrophages (M2 TAMs), while increasing tumor-infiltrating CD4^+^ and CD8^+^ T cells in the tumor microenvironment, indicating that ferroptosis can enhance antitumor immune responses by reversing the immunosuppressive microenvironment [169, 170]. Immunogenic ferroptosis induced by BEBT-908 (PI3K inhibitor) leads to upregulation of major histocompatibility complex I (MHC-I) and activation of endogenous IFN-γ via STAT1, thereby activating the antitumor immune response and enhancing anti-PD1 therapy, effectively inhibits tumor growth [171]. The abnormality of ferroptosis regulator STEAP3 may also be involved in various immune-related signatures in the pathogenesis of HCC [172]. Overall, it is obvious that ferroptosis does have significant immunogenicity and may play a positive role in antitumor immunity.

### Ferroptosis leads to dysfunction of immune-related cells

There are also studies demonstrating that ferroptosis of cancer cells is not immunogenic cell death, despite the release of immunogenicity-related DAMPs and cytokines [173].

On the one hand, ferroptosis may inhibit the survival of antitumor immune cells (including DC, NK, and CD8^+^T cells), resulting in immunodeficiency. Dendritic cells (DCs) are the most effective antigen-presenting cells required for T cell activation and maintenance of T cell-dependent immunity. Recent studies revealed that ferroptosis negatively regulates the maturation of DCs, leads to abnormal antigen-presentation, and inhibits the proliferation and activation of CD8^+^T cells, thereby hindering antitumor immunity in vitro and in vivo [174, 175]. One of the mechanisms is that peroxisome proliferator-activated receptor gamma (PPARG)-mediated ferroptosis of DCs elicits impaired production of cytokines (TNF and IL6), decreased expression of MCH-I, and inhibition of IFN-γ production by CD8^+^T cells, thus driving the formation of an immunosuppressive tumor microenvironment (TME). In addition, normal DCs loaded with ferroptotic cancer cells lose their ability to suppress tumor growth.

In lipid peroxides-rich TME, ferroptosis due to aberrant lipid accumulation reduces the antigen-presentation and antitumor ability of DCs, thereby promoting cancer progression [133, 176, 177]. Ferroptotic cancer cells may significantly induce the dysfunction of CD8^+^T and CD4^+^T cells, and disrupt the maturation and function of DCs, thus reducing the effectiveness of immune checkpoint inhibitor (ICI) therapy [175, 177].

NK cells play the role of antitumor immunity, and the defects of NK cells lead to increased rates of tumorigenesis and progression [178]. Lipid peroxidation-induced ferroptosis inhibits glucose metabolism of NK cells, resulting in NK cell dysfunction, while NRF2 activation alleviates ferroptosis and rescues antitumor activity of NK cells in vivo [179, 180].

Moreover, contrary to previous views, DAMPs released by ferroptotic cells may also exert immunosuppressive effects under certain circumstances. 8-OHG released from ferroptotic pancreatic cells activates the STING-dependent DNA sensor pathway, leading to tumor-associated macrophages (TAMs) infiltration and M2 polarization, promoting Kras-driven tumorigenesis and inflammation-related immunosuppression [162, 181, 182]. Prostaglandin E2 (PGE2) released by ferroptotic cancer cells serves as a critical immunosuppressor that can inhibit the antitumor function of DCs, NK cells, and cytotoxic T lymphocytes [183].

Ferroptosis of T lymphocytes inevitably leads to dysfunction of CD8^+^T and CD4^+^T cells [142, 184]. Lipid peroxide accumulation and ferroptosis caused by GPX4 deficiency impair the ability of T lymphocytes to eradicate viral or parasitic infections [184]. Conversely, overexpression of GPX4 or FSP1 inhibits the ferroptosis of T lymphocytes and maintains their immune effect [185]. In addition, immune checkpoint inhibitors sensitize cancer cells to ferroptosis, which leads to tumor immune escape by directly suppressing the function of immune cells [186, 187]. The fatty acid transporter CD36 enhances the sensitivity of CD8^+^ T cells to ferroptosis by promoting lipid peroxidation, impairing the production of cytotoxic cytokine and antitumor activity. Inhibition of ferroptosis by blocking CD36 restores the antitumor activity of CD8^+^ T cells [187].

On the other hand, some immunosuppressive immune cells, such as anti-inflammatory M2 TAMs and Tregs, require protection against ferroptosis to maintain cell viability, and ferroptosis induction in these cells may reverse their immunosuppressive function [178]. Tregs are essential for maintaining immune tolerance and suppressing antitumor immunity; thus an elevated proportion of Tregs is usually associated with poor prognosis and poor survival in cancer patients. Inhibition of Gpx4 in Tregs leads to lipid peroxides accumulation and ferroptosis, thereby enhancing the efficacy of immunotherapy in tumors with high Treg infiltration [188].

It is reasonable to believe that the immunogenicity of ferroptosis and its impact on antitumor immunity is closely related to tumor stage and TME. Exploring the relationship between ferroptosis and immunity will contribute to developing more effective cancer treatment strategies, and further studies are urgently needed.

### Nanoparticles: ingenious attempts to target the combination of ferroptosis and immunomodulation

**Fig. 4:**
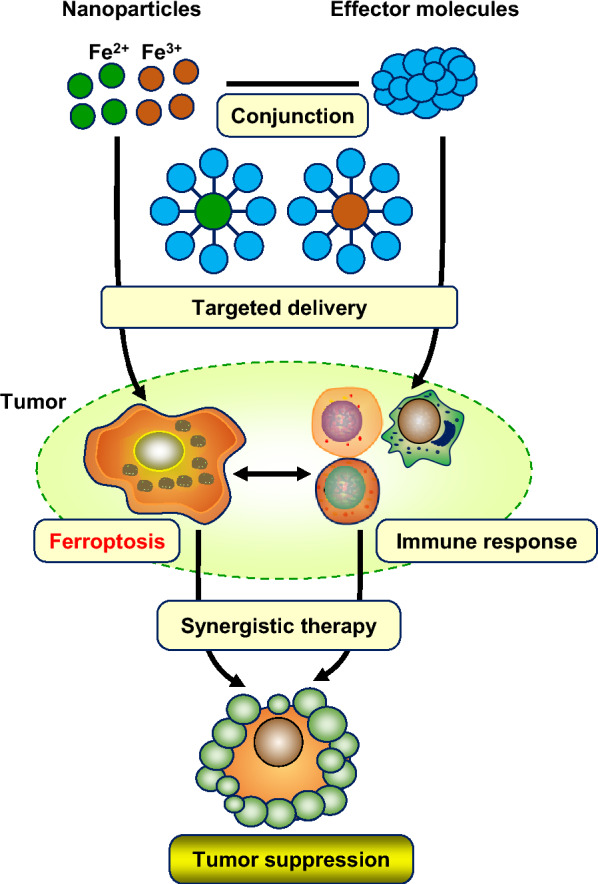
Schematic representation of nanoparticles targeting ferroptosis and immunomodulation. Nanoparticles linked to effector molecules are delivered to tumor targets and simultaneously induce ferroptosis and immunogenetic cell death, thereby enabling ferroptosis-enhanced tumor immunotherapy, and stimulating long-term protective antitumor immune, acting as nano-vaccines

Currently, lots of studies have focused on the synergistic induction of ferroptosis and antitumor immunity by targeting nanoparticles to promote tumor inhibition (Fig. 4). In the mouse 4T1 metastasis model, the biomimetic magnetic nanoparticles Fe_3_O_4_-SAS@PLT trigger ferroptosis by inhibiting system Xc^−^, which induces not only tumor-specific immune responses, but also effectively re-polarize macrophages from immunosuppressive M2 to antitumor M1, improving the efficacy of PD1 immune checkpoint blocking therapy and achieving tumor elimination [189]. Iron oxide-loaded nano-vaccines (IONVs)-mediated tumor microenvironment modification elevates ROS and induces ferroptosis, simultaneously activates M1 macrophages and promotes tumor antigens cross-presentation, completely eradicates aggressiveness and established tumors, and stimulates long-term protective antitumor immunity [190].

In addition to ferroptosis induction, hybrid core–shell vesicles (HCSVs)-induced Fenton reaction exposes calreticulin to tumor cells, leading to maturation of dendritic cells (DC) and tumor infiltration of cytotoxic T lymphocytes, thereby resulting in significant tumor suppression [191]. Ultrasmall single-crystal Fe nanoparticles (BCC-USINPs) simultaneously induce ferroptosis and ICD, promoting DC maturation and triggering adaptive T-cell infiltration by releasing immunogenic DMAPs, enabling ferroptosis-enhanced tumor immunotherapy [192]. Metal-phenolic networks (MPNs) nano-platform synergistically enhances ICD with ferroptosis and promotes primary and distant tumor killing in combination with DNAzyme-mediated PD-L1 inhibition [193]. A study of cancer cell membrane coated RSL3-loaded gold nanocages (C-RAuNC) revealed that RSL3-induced ferroptosis synergistically initiates antitumor immunity with photothermal therapy (PTT)-induced ICD, thereby inhibiting the growth and recurrence of osteosarcoma [194].

Nanoparticles containing ferritin and pH-sensitive molecular switch (FPBC@SN) release sorafenib and NLG919 in acidic cytoplasm. Sorafenib induces ferroptosis by blocking GSH synthesis, down-regulating GPX4, and increasing endogenous iron pools. NLG919 stimulates antitumor immunity by reducing indoleamine-2,3-dioxygenase (IDO)-regulated tryptophan metabolism, synergistically inhibiting cell growth and metastasis of breast cancer in vitro and in vivo [195]. Doxorubicin (DOX) and β-lapachone (LAP) are effective inducers of ICD, and they work synergistically with iron in tumor inhibition. Tumor-targeted delivery of DOX-Fe^2+^ complexes initiates antitumor immunity and induces ICD, effectively integrating tumor metabolism and immune response, exerting a potential antitumor tumor strategy [196, 197]. LAP-Fe^2+^ complexes synergistically trigger ferroptosis and ICD in tumor cells in a remotely controllable manner [198]. Biomimetic magnetosomes composed of Fe_3_O_4_ magnetic nanoclusters, TGF-β inhibitors, and PD-1 antibodies can achieve synergistic cancer treatment through ferroptosis and immunomodulatory [199]. In another study, nanocarriers of exosome inhibitors (GW4869) and Fe^3+^ enhance the response of melanoma to PD-L1 checkpoint blockade both in vivo and in vitro, representing an effective therapeutic strategy*.* Mechanistically, cooperation between GW4869 and Fe^3+^ in nano-units induces ferroptosis and antitumor immune responses against melanoma by reducing exosomes and attenuating exosomal PD-L1, and stimulating cytotoxic T lymphocytes and immune memory [200].

Overall, nanoparticles that simultaneously induce ferroptosis and ICD exhibit great potential in tumor suppression, which may bring new opportunities for the clinical treatment of tumors. However, the in vivo stability, active and passive targeting capabilities of nanoparticles, and their metabolic kinetics and toxicity, require further investigation.

## Conclusions and perspectives

As a novel iron-dependent non-apoptotic RCD, ferroptosis has attracted extensive attention since its discovery. Ferroptosis is deeply involved in the regulation of cellular stress processes such as ER stress and autophagy, and affects the occurrence and development of cancer through a complex and sophisticated signal transduction network. Currently, targeting ferroptosis is emerging as a potentially effective approach for the treatment of various diseases, especially cancer and ischemic organ damage. Fortunately, available evidence suggests that ferroptosis and other known types of RCDs do not appear to be antagonistic to each other, but may coexist and interfere with each other, and act synergistically in cell fate determination. Therefore, the induction of mixed RCD can undoubtedly be an effective therapeutic strategy to break the bottleneck of the limitations of radiotherapy alone. In addition, the reciprocal regulatory feedback between ferroptosis and immune responses may further exert tumor suppressive effects. Furthermore, targeting ferroptosis using small molecule compounds, clinical drugs, and nanoparticles is also a potentially effective antitumor strategy in clinical practice, especially in iron-rich tumors that are prone to ferroptosis.

However, the existing understanding of ferroptosis remains insufficient. First, the execution mechanism of ferroptosis is still unclear. Lethal plasma membrane damage due to lipid peroxidation is the direct cause of ferroptosis; however, whether other unknown downstream molecules exist to directly regulate and execute ferroptosis, like a role BCL family proteins or Caspase-3 served for apoptosis, is still elusive. Therefore, cracking the execution mechanism of ferroptosis remains one of the critical issues in the next few years.

Second, the selective mechanism of ferroptosis remains poorly understood. At present, it is still at a loss when mentioned to selectively activate or inhibit ferroptosis in specific tissues and cells in different diseases. Whether pharmacological intervention can selectively inhibit tumor cells without causing severe toxic side effects on normal cells remains to be verified. Comprehensively understanding the susceptibility mechanism of cancer cells and immune cells to ferroptosis, selectively inhibiting or promoting ferroptosis of immune cells, and creating an antitumor immune microenvironment are still important issues to be solved urgently.

Third, definitive biomarkers for ferroptosis have not been identified. The search for predictive clinical biomarkers of ferroptosis, especially those that can be directly and rapidly detected in patient body fluids and biopsy samples, will be of fundamental importance in determining the physiological function and therapeutic potential of ferroptosis. Thus, finding biomarkers to facilitate the accurate detection of ferroptosis will also be an active area of research in the coming years.

Fourth, the clinical translation potential of targeted ferroptosis therapy needs to be validated. Based on the results obtained so far, approaches that combine FINs with other therapies, such as radiotherapy and immunotherapy, to improve the treatment effect have great potential for clinical translation. Unfortunately, most FINs exhibit poor pharmacology and pharmacokinetics in animal models, which directly limits their clinical application. Existing medicines, such as Sorafenib, Lapatinib, Sulfasalazine, Cisplatin, etc., can effectively induce ferroptosis [79, 201–203]. However, a recent study showed that sorafenib is not qualified as an effective ferroptosis inducer, and ferroptosis induced by system Xc^−^ inhibitor can only be achieved in a subset of tumor cell lines [204]. Thus, under the premise of rigorous evaluation, innovative uses of these medicines in combination with other therapies to target ferroptosis may acquire great potential in specific types of tumor therapy. In addition, nanoparticles coated with specific ligands and carrying targeted drugs also have good potential for clinical translation. However, the stability, targeting capabilities, kinetics, and toxicity of nanocarriers still need to be intensively studied in vivo.

Addressing these unanswered questions promises to yield new insights, not only for ferroptosis research itself, but also for the biomedical frontiers associated with this novel RCD. It is believed that in the next few years, there will be exciting discoveries in the field of ferroptosis-related research. Based on specific biomarkers and precise patient-specific assessment, targeting ferroptosis will be translated into a practical new approach to clinical cancer therapy, significantly contributing to the prevention, diagnosis, prognosis, and treatment of cancer.

## Data Availability

Not applicable.

## Abbreviations

8-OHG: 8-Hydroxyguanosine
ACSL4: Acyl-CoA synthetase long-chain family member 4
ALOXs: Lipoxygenases
AML: Acute myeloid leukemia
ATM: Ataxia telangiectasia mutated protein
BH4: Tetrahydrobiopterin
cGAS: Cyclic GMP–AMP synthase
CoQ_10_: Coenzyme Q_10_
DAMPs: Damage-associated molecular patterns
DHODH: Dihydroorotate dehydrogenase
DMT1: Ferrous ion membrane transport protein 1
FPN: Ferroportin
FSP1: Ferroptosis suppressor protein 1/AIFM2
GCH1: GTP cyclohydrolase 1
GCL: Glutamate–cysteine ligase
GGT1: Gamma-glutamyltransferase 1
GSS: Glutathione synthase
GPX4: Glutathione peroxidase 4
GR: Glutathione reductase
GSH: Glutathione
GSSG: Oxidized glutathione disulfide
HLF: Hepatic leukemia factor
HMGB1: High mobility group protein B1
IFNγ: Interferon-gamma
KRAS (G12D): Kirsten rat sarcoma viral oncogene homolog-G12D mutation
LPCAT3: Lysophosphatidylcholine acyltransferase 3
MVBs: Multivesicular bodies
PD-L1: Programmed cell death-ligand 1
PERK: PKR-like ER kinase
POR: Cytochrome P450 oxidoreductase
PROM2: Prominin-2
PUFA: Polyunsaturated fatty acid
PUFA-PLs: Polyunsaturated fatty-acid-containing phospholipids
PUFA-PL-OH: PUFA phospholipid alcohols
PUFA-PL-OOH: Phospholipid with peroxidized polyunsaturated fatty acyl tail
ROS: Reactive oxygen species
RT-MPs: Irradiated tumor cell-released microparticles
STEAP3: Six-transmembrane epithelial antigen of the prostate 3
STING: Stimulator of interferon genes protein
TFRC: Transferrin receptor
TGFβ: Transforming growth factor β
TNBC: Triple negative breast cancer
TNFα: Tumor necrosis factor-alpha
